# Soft viscoelastic properties of nuclear actin age oocytes due to gravitational creep

**DOI:** 10.1038/srep16607

**Published:** 2015-11-18

**Authors:** Marina Feric, Chase P. Broedersz, Clifford P. Brangwynne

**Affiliations:** 1 Department of Chemical and Biological Engineering, Princeton University, Princeton, NJ 08544, USA; 2 Lewis-Sigler Institute for Integrative Genomics and Joseph Henry Laboratories of Physics, Princeton University, Princeton, NJ 08544, USA

## Abstract

The actin cytoskeleton helps maintain structural organization within living cells. In large *X. laevis* oocytes, gravity becomes a dominant force and is countered by a nuclear actin network that prevents liquid-like nuclear bodies from immediate sedimentation and coalescence. However, nuclear actin’s mechanical properties, and how they facilitate the stabilization of nuclear bodies, remain unknown. Using active microrheology, we find that nuclear actin forms a weak viscoelastic network, with a modulus of roughly 0.1 Pa. Embedded probe particles subjected to a constant force exhibit continuous displacement, due to viscoelastic creep. Gravitational forces also cause creep displacement of nuclear bodies, resulting in their asymmetric nuclear distribution. Thus, nuclear actin does not indefinitely support the emulsion of nuclear bodies, but only kinetically stabilizes them by slowing down gravitational creep to ~2 months. This is similar to the viability time of large oocytes, suggesting gravitational creep ages oocytes, with fatal consequences on long timescales.

The mechanical properties of living cells are important for their ability to maintain structural integrity and withstand a variety of physiological forces. For example, the mechanical properties of red blood cells determine how easily they can bend and deform without rupturing as they move through the vasculature^1^. The periodic stretching of the cardiovascular system leads to cycles of deformation of cells such as cardiac myocytes and lung epithelia; dysregulation of the mechanical response of these cells is at the root of numerous pathologies^2^,^3^,^4^,^5^. The mechanical properties of cells are, in turn, strongly influenced by their local mechanical environment, and can influence their differentiation programs^6^. The material properties of cells and their components are thus adapted to generate and withstand forces involved in numerous biological functions.

The primary mechanochemical structure in animal cells is the cytoskeleton, a load-bearing mechanical scaffold, which provides shape and structure to the cell. Actin is a key cytoskeletal biopolymer and is one of the primary cellular components controlling the cell’s mechanical properties. Work on numerous cell types has shown that the cytoskeleton is viscoelastic: moduli are typically on the order of 0.1 kPa–100 kPa^7^,^8^,^9^,^10^,^11^, properties that are strongly dependent on polymerized actin. To isolate actin’s individual contribution, much work has been done to characterize *in vitro* reconstituted networks consisting of purified actin polymerized into networks with defined structure and composition^12^,^13^,^14^,^15^. These studies reveal that purified actin networks are viscoelastic materials whose properties are highly sensitive to the amount and type of actin binding proteins, as well as the degree of motor activity^16^,^17^,^18^,^19^.

In addition to providing mechanical support for cells bearing externally applied loads, the actin cytoskeleton also plays numerous roles in maintaining intracellular organization. For example, cells coordinate actomyosin contractility with force generation by the microtubule spindle to faithfully segregate DNA and other cell components during cell division^20^,^21^,^22^. Interestingly, large starfish oocytes accomplish chromosome segregation using actin in novel ways^23^. Actin and tubulin are also involved in nuclear positioning, for instance to maintain polarity, by either firmly anchoring the nucleus or by actively moving it^24^,^25^,^26^. Positioning of cell surface molecules is also directly affected by remodeling of the actin cortex^27^,^28^, while organelle localization and transport within the cell can be strongly influenced by the actin cytoskeleton^29^,^30^.

Actin’s primary mechanical functions have been well-described in the cytoplasm, but actin is also present in the nucleus, where it could potentially serve as a mechanical scaffold^31^,^32^,^33^,^34^. In somatic cells, actin concentrations are typically low; however, large oocytes of *Xenopus laevis* have evolved to specifically maintain high concentrations of nuclear actin^35^. This nuclear actin forms a three dimensional meshwork that stabilizes embedded nuclear bodies against gravitational sedimentation^36^; these nuclear bodies represent liquid phases of RNA/protein organelles (RNP bodies)^37^, and actin disruption leads to their large scale sedimentation and coalescence^36^ (Fig. 1). Several actin binding proteins have recently been found within the nucleus (germinal vesicle, GV), but why and how *X. laevis* controls nuclear actin mechanics is still unclear^38^. Furthermore, nothing is known about the mechanical properties of this nuclear actin network, nor how these properties allow it to physically support the contents against gravitational forces during the process of cell growth (oogenesis) within the ovary.

## Asymmetric Organelle Distributions

**Figure 1:**
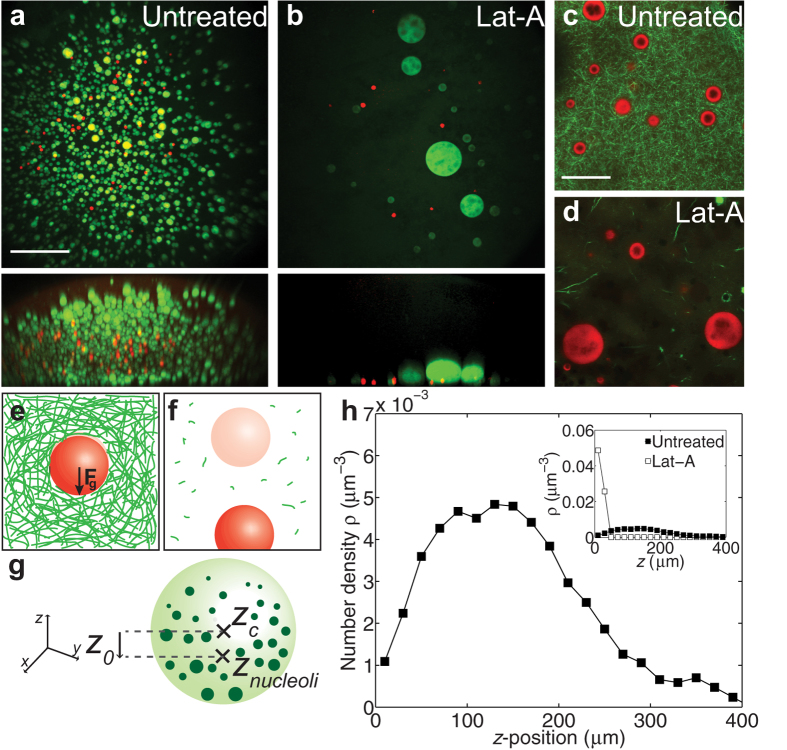
Non-uniform distribution of nuclear bodies is dependent on a nuclear actin meshwork. (**a,b**) Top images are maximum intensity XY projections of the entire nucleus containing nucleoli labeled with NPM1::GFP (green) and HLBs labeled with TagRFP::coilin (red); bottom images are maximum intensity XZ projections. Scale bar = 100 μm. (**a**) An emulsion of nuclear bodies is stabilized by a nuclear actin network. (**b**) A few massive nuclear bodies are found at the bottom 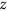-plane after actin disruption by Lat-A. (**c,d**) XY views of Lifeact::GFP labeled actin network (green) and NPM1::RFP labeled nucleoli (red) in untreated (**c**) and Lat-A treated nuclei (**d**). Scale bar = 20 μm. (**e**) Schematic showing how a nuclear body (red) is subjected to a downward gravitational force, but is held in place by an actin network (green). (**f**) Disruption of the actin network results in nuclear body sedimentation. Faded sphere represents initial position. (**g**) Schematic illustrating the distance, *z*_*0*_, as the difference from the center of the nucleolar distribution, *z*_*nucleoli*_, and the centroid of the nucleus, *z*_*c*_. (**h**) The normalized number density, as a function of vertical z-position, where the lowest 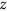-position, 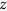 = 0, corresponds to the bottom of the native nucleus (n = 43 nuclei). Inset contains comparison of normalized number density as a function of 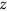-position for untreated nuclei (filled symbols) and Lat-A treated nuclei (unfilled symbols) (n = 12 nuclei).

To investigate how actin supports nuclear bodies against gravity, we visualized nucleoli and histone locus bodies (HLBs), using nuclei expressing NPM1::GFP and TagRFP::coilin fluorescent constructs, respectively, together with multiphoton microscopy (Fig. 1a). Three-dimensional imaging of the nucleus revealed that these nuclear bodies are suspended throughout the nucleus. Visualization of the actin network using Lifeact::GFP confirms that the nuclear bodies are larger than the mesh size of the network, and thus their motion is highly constrained (Fig. 1c). However, their spatial distribution is typically highly asymmetric, with the majority of nuclear bodies concentrated towards one end of the nucleus (Fig. 1h). To quantify this asymmetry, we used custom image analysis to obtain the apparent gravitational (*z*) axis (Methods) and then determined the average position of nucleoli, 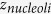, and the centroid position of the nucleus, 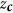; this defines a distance, 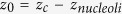 that quantifies the deviation of the spatial distribution of nuclear bodies from the center of the nucleus (Fig. 1g, Methods). We determined the number density profile along this *z*-axis, yielding a non-uniform distribution of nuclear bodies with a deviation of 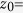 50 ± 20 μm (mean ± s.d.) from the center of the nucleus (Fig. 1h).

Consistent with our previous work, actin disruption using latrunculin-A (Lat-A) (Fig. 1h inset) gives rise to significant gravitational sedimentation and coalescence of nucleoli at the bottom of the nucleus (Fig. 1b,d)^39^; HLBs are significantly fewer and less dense than nucleoli, but also undergo sedimentation and coalescence. These data show that nuclear actin is necessary for keeping the nuclear bodies distributed in three dimensions. However, how the actin meshwork mechanically controls the spatial distribution of nuclear bodies, and what causes the asymmetric distribution in the unperturbed nucleus remain unclear (Fig. 1e,f).

## Active Microrheology Reveals Soft Viscoelastic Nature of Nuclear Actin

**Figure 2:**
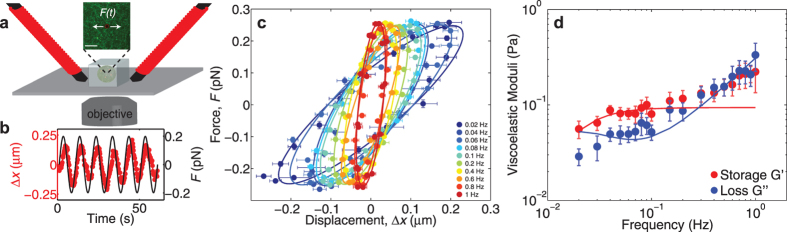
Nuclear actin forms a soft, viscoelastic network. (**a**) Schematic diagram of active microrheology experimental set-up with two opposing electromagnets. Inset shows XY image of Lifeact::GFP labeled actin network (green) with an embedded magnetic microsphere (red, R = 1.5 μm). Scale bar = 10 μm. (**b**) An example of sinusoidal applied force (black) and response of magnetic microsphere (red). (**c**) Lissajous (stress-strain) plot of applied magnetic force as a function of measured displacement of microspheres in one nucleus. Error bars are s.e.m. Curves are fit to an ellipse and color denotes applied frequency. (**d**) Viscoelastic moduli, storage modulus (red) and loss modulus (blue), as a function of frequency in the untreated nucleus (n = 9 nuclei). Error bars represent s.e.m. Solid line is from fit determined by viscoelastic model in Fig. 3 inset.

To determine how the mechanical properties of nuclear actin are related to its role in supporting nuclear bodies, we directly measured these mechanical properties using active microrheology. Using custom-built electromagnetic tweezers, we applied oscillatory forces on magnetic microspheres injected into the nucleus (Fig. 2a); the resulting periodic bead displacements were measured using sub-pixel resolution particle tracking algorithms (Fig. 2b)^40^. Plots of the applied force versus displacement, known as Lissajous curves^41^, exhibit an elliptical shape whose orientation changes with frequency, consistent with a linear viscoelastic response in this force range (Fig. 2c). We used this data to determine the storage modulus, 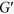, and the loss modulus, 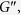 over a range of frequencies from 0.02 Hz–1 Hz. Remarkably, we find the network to be viscoelastic and surprisingly soft, with storage and loss moduli on the order of 0.1 Pa (Fig. 2d); this is several orders of magnitude lower than the moduli typically measured in the cytoplasm, but notably similar to networks of entangled actin filaments *in vitro*^12^,^42^,^43^,^44^,^45^.

**Figure 3:**
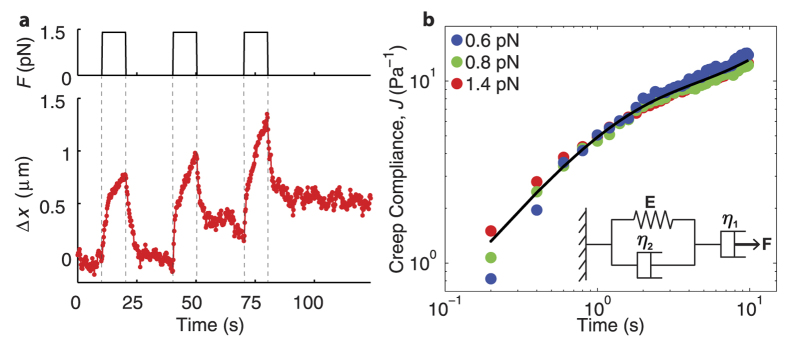
Nuclear actin undergoes creep under constantly applied stress. (**a**) Creep response for an individual bead at a force of 1.4 pN. Top shows applied force, and bottom shows displacement. (**b**) Averaged creep compliance as a function of time for different applied force (blue = 0.6 pN, green = 0.8 pN, and red = 1.4 pN) for the untreated nucleus, where n = 6, 4, and 7 nuclei, respectively. Black line represents fit for combined data based on viscoelastic model. Inset shows schematic of extended Kelvin-Voigt model.

To further probe the viscoelastic behavior of this nuclear actin network, we applied step pulses of force. During periods of constant force, 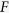, we observe that the network does not simply displace by a constant amount, 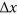, as would be expected for a pure solid, but instead continues to deform in time (Fig. 3a); such creep behavior is typical of viscoelastic materials. The creep compliance, 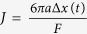, can be well-fit by a simple viscoelastic Kelvin-Voigt model in series with a dashpot, representing long-time flow due to network rearrangements (Fig. 3b inset); fits yield values for the model parameters, E = 0.14 ± 0.01 Pa, η_2_ = 0.15 ± 0.01 Pa-s, and η_1_ = 1.7 ± 0.2 Pa-s (95% confidence interval), with the latter reflecting the long time network viscosity. Moreover, the creep compliance is nearly identical even when the force magnitude is varied, consistent with a linear response (Fig. 3b). Within the linear response regime, the creep compliance can be related to the oscillatory force response—i.e. 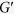 and 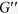 (see Methods). Indeed, we find that the simple viscoelastic model also fits to the oscillatory response, yielding roughly similar model parameters (E = 0.10 ± 0.01 Pa, η_2_ = 0.05 ± 0.01 Pa-s, and η_1_ = 0.9 ± 0.3 Pa-s, 95% confidence interval) (Fig. 2d); this confirms the consistency between the creep and oscillatory data, and further supports the use of the simple Kelvin-Voigt type viscoelastic model in describing the viscoelastic behavior of nuclear actin.

## Dependence of Spatial Distribution and Organelle Size on Gravitational Force

**Figure 4:**
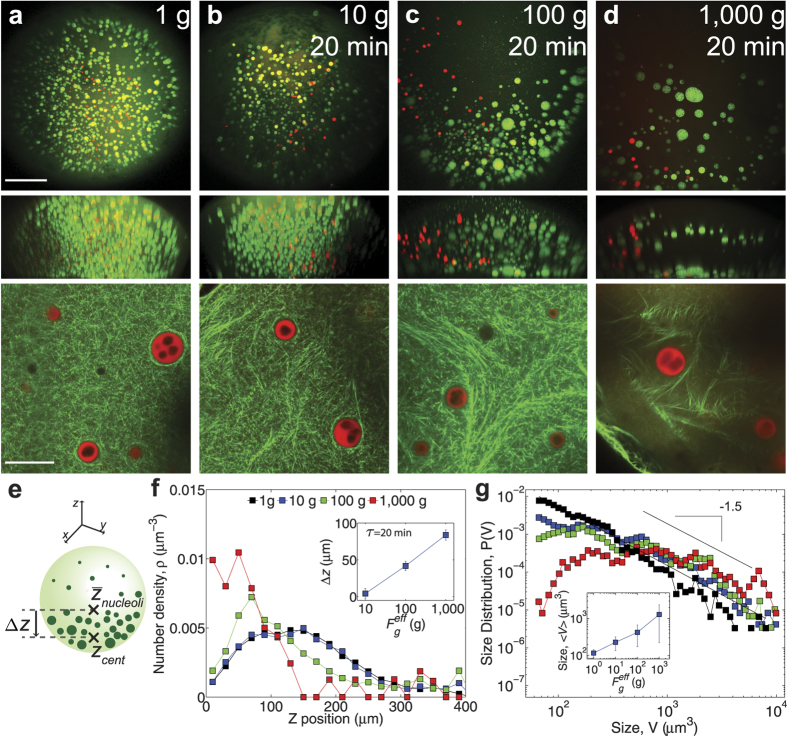
Centrifugation changes nuclear body spatial and size distributions in a force dependent manner. (**a**–**d**) Top images are maximum intensity XY projections of the entire nucleus containing nucleoli labeled with NPM1::GFP (green) and HLBs labeled with TagRFP::coilin (red); middle images are maximum intensity XZ projections of the same nucleus; and bottom images are XY views of the Lifeact::GFP labeled actin network with NPM1::RFP labeled nucleoli. For top and middle rows, scale bar = 100 μm, and for bottom row, scale bar = 20 μm. (**a**) The untreated nucleus contains suspended nuclear bodies at 1 g. For (**b–d**), the nucleus was centrifuged at 10 g (n = 21 nuclei), 100 g (n = 22 nuclei), or 1,000 g (n = 15 nuclei), for twenty minutes, respectively and nuclear bodies and nuclear actin were subsequently imaged. (**e**) Schematic illustrating relative displacement, Δz, between the average center of the nucleolar distribution in the native nucleus, 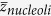, and the average center of the nucleolar distribution after centrifugation, 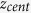. (**f**) The normalized number density, as a function of vertical 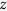-position of nucleoli, where the lowest 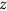-position, 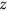 = 0, corresponds to the bottom of the nucleus for untreated nuclei (black) and nuclei centrifuged at 10 g (blue), 100 g (green), and 1,000 g (red) for twenty minutes. Inset shows the relative sedimentation displacement, Δz, as a function of the applied gravitational force. Error bars represent s.e.m. (**g**) The normalized probability distribution of nucleolus size (volume) for untreated nuclei (black) and nuclei centrifuged at 10 g (blue), 100 g (green), and 1,000 g (red) for twenty minutes. Solid line shows a slope of −1.5, indicative of power-law observed in native nuclei. Inset shows the average size of nucleoli vs. applied gravitational force. Error bars represent s.e.m.

The microrheology data show that if particles embedded within this nuclear actin network are subjected to a constant force, they slowly move as the network undergoes continuous creep deformation. Since nuclear bodies are subjected to the constant force of gravity (Fig. 1e), we reasoned that they could be slowly moving downwards, even when embedded within an intact actin network. However, since these forces are relatively small, 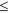
0.1 pN, the resulting creep response may be difficult to observe on experimentally accessible timescales. To determine if nuclear bodies could indeed undergo creeping motion, we subjected the nucleus, with unperturbed nuclear actin, to increased gravitational forces via centrifugation; higher centrifugation rates increase the effective gravitational constant, and thus increase the force on the nuclear bodies. Imaging the actin network using Lifeact::GFP reveals that nuclear actin is largely intact during these centrifugation experiments, up to ~1,000 g where significant disruption of the actin network becomes apparent (Fig. 4a–d bottom).

By centrifuging for twenty minutes at 10 g, 100 g, and 1,000 g (Fig. 4b–d), we find that the spatial distribution of the nuclear bodies greatly depends on applied force (Fig. 4f). While there is only a small effect at 10 g, centrifugation at 100 g and 1,000 g causes the spatial distribution of nucleoli to be highly asymmetric, reflecting significant sedimentation of nucleoli. We quantify this asymmetry by defining the relative sedimentation displacement, 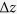, as the distance between the average 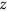-position of nucleoli in the centrifuged nucleus, 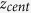, and the average 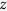-position of the nucleoli in the native nucleus, 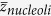 : 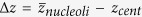 (Fig. 4e, Methods). After twenty minutes of centrifugation, 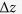
increases with the effective gravitational force (inset Fig. 4f).

Increasing the effective gravitational force by centrifugation also has consequences for nuclear body size. Consistent with our previous measurements, the size distribution of nucleoli in the native nucleus (i.e. 1 g) follows a power-law size distribution, 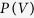 ~ *V*^a^, with an exponent 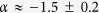 (Fig. 4g); a power law distribution with an exponent of 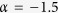 is expected for droplet generation and coalescence over long timescales^46^. We find that the average size of nucleoli steadily increases with increased effective gravitational force (Fig. 4g inset). Consistent with this, we begin to observe a deviation from the power-law size distribution for higher forces: the distribution becomes more flat, reflecting a depletion of smaller nucleoli due to coalescence events (Fig. 4g). Thus, for a fixed centrifugation time (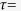
20 min) increasing the effective force of gravity leads to significant disruption of nuclear organization. Indeed, at the highest forces (1,000 g), the distribution of nucleoli is reminiscent of the complete sedimentation of nucleoli observed upon pharmacological disruption of the actin network (Fig. 1b,h inset).

**Figure 5:**
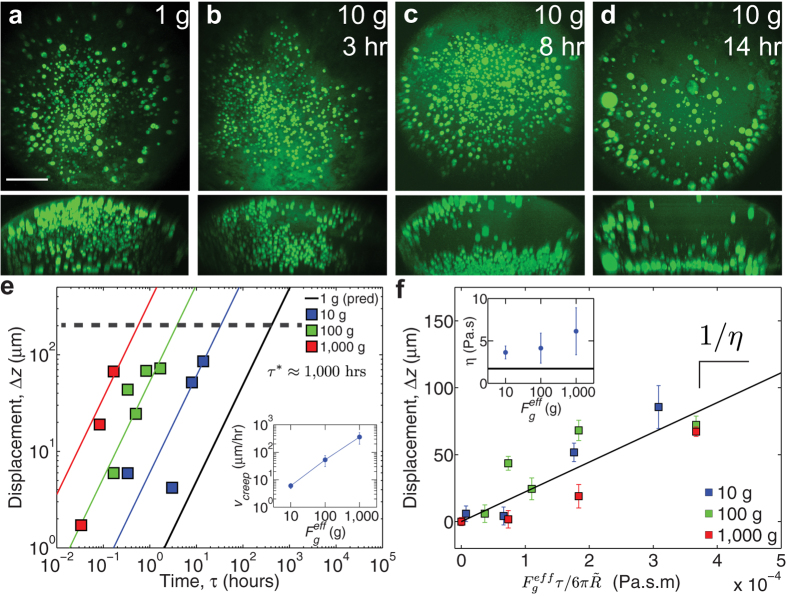
Spatial distributions evolve in time, consistent with creep behavior. (**a**–**d**) Top images are maximum intensity XY projections of the entire nucleus containing nucleoli labeled with NPM1::GFP (green); bottom images are maximum intensity XZ projections of same nuclei. Scale bar = 100 μm. (**a**) The untreated nucleus at 1 g. For (**b–d)**, the nucleus was centrifuged at 10 g for 3 hours (n = 14 nuclei), 8 hours (n = 9 nuclei), and 14 hours (n = 9 nuclei), respectively. (**e**) The relative sedimentation displacement, Δz, as a function of centrifugation time at centrifugation forces of 10 g (blue), 100 g (green), and 1,000 g (red). Error bars represent s.e.m. Black solid line presents prediction at 1 g using Stokes law and average viscosity determined from centrifugation experiments. Dashed line represents a distance of 200 μm, representing the radius of the nucleus. Inset shows the creep velocity as function of effective gravitational force for 10 g, 100 g and 1,000 g. Error bars represent 95% confidence interval from the fit. (**f**) The relative sedimentation displacement, Δz, as a function of the scaled force for centrifugation forces of 10 g (blue), 100 g (green), and 1,000 g (red). Error bars represent s.e.m. Black solid line is the best-fit line for the data, and the slope is the inverse of the viscosity. Inset shows the viscosity individually determined for effective gravitational forces with error bars representing 95% confidence interval. Black solid line indicates the viscosity determined from the creep compliance fit by the viscoelastic model (Fig. 3b).

To gain further insight into the mechanics of nuclear body creep, we monitored the time-dependence of the average position of nucleoli under different centrifugation speeds; these centrifugation speeds correspond to different effective gravitational forces: 
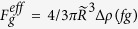
, where 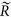 is the median nucleolar size, 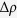 is the density difference between nucleoli and the surrounding nucleoplasm, which we previously measured^36^, and 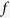 is the relative 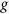 force. Consistent with the creep response observed in our microrheology experiments (e.g. Fig. 3), we find that under a given gravitational force, the apparent sedimentation distance, 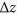, increases with increasing centrifugation time, 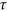 (Fig. 5a–e). This displacement allows us to determine a creep velocity, 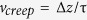, as a function of applied force (Fig. 5e). Consistent with the large displacements observed for higher forces (Fig. 4), 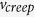 increases in direct proportion to increasing force, 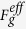
(Fig. 5e inset).

Based on these observations, together with our microrheology data showing that the actin network exhibits viscous behavior on long time scales, we model the creep dynamics of nucleoli using Stokes law: 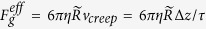. In agreement with Stokes law, the creep data could be collapsed onto a single curve, by plotting the relative sedimentation displacement, 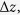 as a function of a scaled force-time variable: 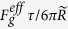, using the median nucleolar size, 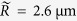 (Fig. 5f). The inverse of the slope of these data is the apparent long-time viscosity of the nucleoplasm, 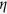 ∼ 1 Pa-s (Fig. 5f inset), which reflects the relaxation of nuclear actin filaments. For all centrifugation conditions, we obtain similar viscosities (within error), suggesting that the material properties of the actin network are not strongly impacted during centrifugation. These data are quantitatively consistent with the long-time viscosity obtained from either the creep response, 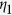 = 1.7 ± 0.2 Pa-s (Fig. 3b), or from oscillatory microrheology 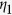 = 0.9 ± 0.3 Pa-s (Fig. 2d).

## Modeling Gravitational Creep Under Native Conditions

The ability to scale all the data using a single force-time variable, 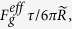 shows that the gravitational force and elapsed time are intrinsically linked (Fig. 5f). For large forces (e.g. 1,000 g), the spatial distribution of nuclear bodies looks similar on short time scales as the low-force data (e.g. 10 g) on long time scales. This raises the question: under natural conditions of a constant low gravitational force of 1 g, how long would it take for gravitational creep to cause complete sedimentation of nuclear bodies? Structural organization within the nucleus would be severely disrupted when the apparent sedimentation distance, 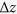, is comparable to the radius of the nucleus, i.e. when 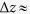
200 μm. The timescale for severe disruption of nuclear architecture by gravitational creep goes from roughly 1 hour to 100 hours as the effective gravitational force goes from 1,000 g to 10 g. In a native nucleus under 1 g, the timescale, τ^*^, for gravitational creep to disrupt nuclear architecture is thus on the order of τ^*^ ≈ 1,000 hours, or ~1 month.

**Figure 6:**
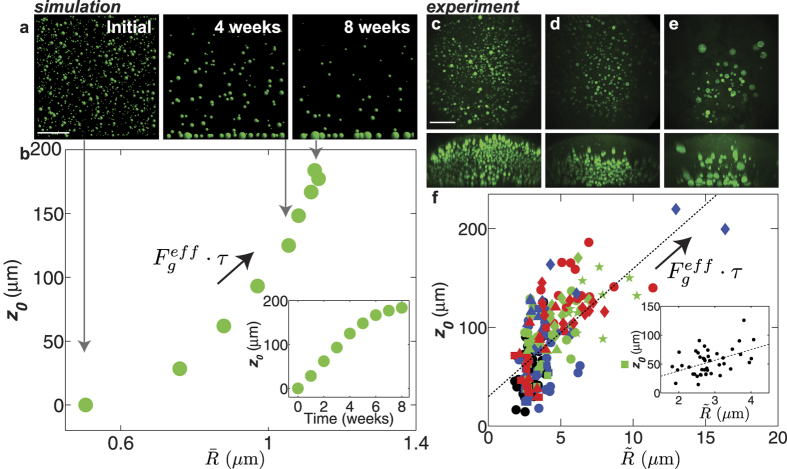
Signatures of aging due to gravitational creep. (**a**) Brownian dynamics simulation results (XZ plane) of gravitational creep over time. Scale bar = 100 μm. (**b**) The correlation between the distance, 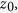 of the nucleoli from the center of the simulation box compared to the mean nucleolar size. Inset shows the distance, 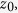 of the simulated nuclear bodies from (**a**) as a function of time. (**c–e**) Top images are maximum intensity XY projections of the entire (untreated) nucleus containing nucleoli labeled with NPM1::GFP (green); bottom images are maximum intensity XZ projections. Scale bar = 100 μm. From left to right, the spatial asymmetry of nuclear bodies becomes more pronounced and the average size of nuclear bodies increases. (**f**) The distance, 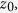 of the center of the nucleolar distribution from the center of the nucleus compared to the median nucleolar size for all the centrifugation data combined; dashed black line is the best-fit line (correlation coefficient, ρ = 0.67). Arrow points in the direction of increasing effective force, 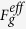, and/or centrifugation time, 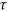. Inset shows the correlation between the distance, 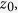 and median nucleolar size for only the native data; dashed black line is the best-fit line (correlation coefficient, ρ = 0.46). Symbols are: native 1 g (black circles, n = 43 nuclei), 10 g 20 min (blue circles, n = 21 nuclei), 10 g 3 h (blue squares, n = 14 nuclei), 10 g 8 h (blue triangles, n = 9 nuclei), 10 g 14 h (blue diamonds, n = 9 nuclei), 100 g 10 min (green squares, n = 9 nuclei), 100 g 20 min (green circles, n = 22 nuclei), 100 g 30 min (green triangles, n = 9 nuclei), 100 g 50 min (green diamonds, n = 9 nuclei), 100 g 100 min (green pentagons, n = 12 nuclei), 1,000 g 2 min (red squares, n = 11 nuclei), 1,000 g 5 min (red triangles, n = 11 nuclei), 1,000 g 10 min (red diamonds, n = 11 nuclei), and 1,000 g 20 min (red circles, n = 15 nuclei).

To illustrate this surprising prediction, we developed a simple Brownian dynamics simulation of nuclear bodies moving in a viscoelastic meshwork. The creep behavior in the simulation is defined by the experimentally-determined long time viscosity of the network, 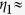
2 Pa-s (Fig. 6a). The simulation begins with randomly distributed nuclear bodies with a power law size distribution, 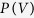    ~ *V*^−1.5^. The bodies are subjected to fluctuating Brownian forces, as well as a constant gravitational force, and fuse when they collide. Similar to our experimental observations, nuclear bodies undergo significant sedimentation, by an amount 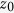, which for early times depends linearly on the total time elapsed (Fig. 6b inset). Moreover, consistent with the extrapolation of our experimental data, these simulations also predict that under native conditions of 1 g, nuclear bodies have also undergone nearly complete sedimentation on a timescale of 1–2 months.

## Signatures of Viscoelastic Aging in Native Oocytes

These simulations suggest that younger oocytes, which would have been subjected to gravitational creep for shorter periods of time, should have a more uniform spatial distribution of nuclear bodies. Furthermore, the younger oocytes would tend to have smaller nucleoli due to fewer coalescence events, compared with older ooctyes (Fig. 6b). Oogenesis is an asynchronous growth process that occurs within the ovary of the frog, making it difficult to precisely determine the exact age of mature oocytes^47^. However, we observed that the degree of asymmetry of the spatial distribution and typical size of the nuclear bodies is highly variable, consistent with variable oocyte age (Fig. 6c–e).

In both our simulations (Fig. 6b) and centrifugation experiments (Fig. 6f), we observe a strong correlation between the degree of asymmetry (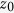) and the median nucleolar size, 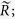 for centrifugation experiments, the correlation coefficient is ρ = 0.67, with 95% confidence bounds 0.59 ≤ ρ ≤ 0.74 (Fig. 6f). This correlation arises since both of these parameters are strongly dependent on the force-time variable, 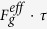. Consistent with a gravity-induced viscoelastic aging process, we also find a statistically significant correlation in the native nucleus (inset Fig. 6f; correlation coefficient, ρ = 0.46, 0.19 ≤ ρ ≤ 0.67). Taken together with our microrheology data, the long timescale Stokes model and simulations, our data provide strong support for the conclusion that gravitational creep occurs within the developing oocyte.

## Discussion

The frog *X. laevis* has evolved a nuclear actin network whose primary function is to support the nuclear contents against gravity. Here, we have investigated the material properties of this network, revealing a surprisingly soft viscoelasticity, comparable to entangled actin networks *in vitro*. The creep response, which we quantify for embedded probe particles and endogenous nuclear bodies, underscores the viscous response of this actin network on long timescales. Similar creep responses to exogenous force application have previously been described for living cells, for example with attached magnetic particles subjected to constant force pulses^7^, or with cells stretched between two microplates^48^. However, viscoelastic moduli in living cells are typically much higher, with values reported from 1 Pa up to the kPa range^11^,^37^, compared with the ~0.1 Pa moduli we observe for this nuclear actin network. This suggests that nuclear actin is only very weakly cross-linked, if at all.

The surprisingly soft viscoelastic properties we report here could be a generic feature of nuclear actin networks. Indeed, to our knowledge, our study is the first to precisely quantify the viscoelastic properties of an actin network within the cell nucleus. While actin appears to be present in the nucleus of most cells^31^,^33^, its concentration tends to be relatively low in typical somatic cells, and the degree to which actin polymerizes into a load-bearing scaffold is unclear. However, in the large oocyte of *X. laevis*, actin is maintained at high concentrations within the nucleus^35^, and this actin is polymerized into a scaffold with a mesh-size of roughly ~0.5 μm^36^. Thus, actin appears to assemble into only very weak viscoelastic networks within the nucleus, and in some somatic nuclei may not even form a connected meshwork.

The key difference between the oocyte nuclei we study here, and those of somatic cells, is their size: *X. laevis* oocytes grow to be over 1 mm in diameter, where the physics of gravitational sedimentation become increasingly important^36^. The primary consequence of disrupting nuclear actin is the immediate sedimentation and large-scale coalescence of liquid-like RNP droplets, including nucleoli and histone-locus bodies. This sedimentation and coalescence into a small number of very large organelles is likely to be highly disruptive to the function of these organelles. The nuclear actin network in these ooctyes thus appears to have evolved to kinetically stabilize the nuclear contents against these gravitational forces, by dramatically slowing down disruptive viscoelastic creep displacements. This is consistent with the observed power-law size distribution of nucleoli, 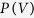 ~ *V*^−1.5^, which arises from slow coarsening dynamics due to viscoelastic relaxations that occur during the long period of oogenesis. We note that even in the absence of gravity, nucleolar droplets would still coarsen due to long-time diffusive motion and rearrangements in the network. However, gravity appears to accelerate droplet coarsening, by biasing their direction of motion downwards, causing increased droplet interactions and coalescence.

The actin network stabilizes nuclear bodies on short time scales, but our data show that by 1–2 months, gravitational creep within the oocyte will lead to severe disruption of nuclear organization; this will have a significant impact on oocyte viability (Fig. 6). Consistent with this, large mature (Stage VI) *X. laevis* oocytes only remain viable within the ovary for roughly 1–3 months, before being resorbed in a process known as atresia^47^,^49^,^50^. Thus, the weak viscoelastic properties of this nuclear actin network appear to limit the lifetime of oocytes. Viscoelasticity is a ubiquitous feature of the cytoskeleton, and gravitational creep could similarly affect the organization of other large cells. Gravity could therefore also disrupt intracellular organization and impact the viability of large human oocytes, which may be related to the timing of atresia^51^. Indeed, gravitational creep likely plays an important role in cellular growth and aging in many large cells.

## Methods

### Oocyte collection

Frogs were anesthetized with 0.1% MS-222 solution for 15 minutes, and oocytes were surgically removed from adult female *X. laevis* frogs. Oocytes were incubated at 18 °C in OR2 solution. To remove the follicular layer, the oocytes were first mechanically separated and then incubated for 1 hour and 20 minutes in 2 mg/ml collagenase (Sigma). Stage V-VI oocytes of diameter of 1–1.3 mm were used for all experiments and identified using a Zeiss stereoscope^47^. All methods were carried out in accordance with approved guidelines set by the Institutional Animal Care and Use Committee (IACUC) for animal use for research purposes. All experimental protocols were approved by IACUC (protocol #1839-14) at Princeton University.

### DNA and mRNA constructs

All constructs used were based on pCS2 + vector backbones. NPM1::eGFP, NPM1::RFP and Lifeact::GFP were used as previously described^36^. TagRFP::coilin was cloned from a vector containing TagRFP, a gift from Jens Bernhard Bosse, and a vector containing GFP::coilin, a gift from Joseph Gall by recombining TagRFP and coilin into a pCS2 + vector. An SP6-based capped RNA transcription kit was used to make mRNA from the linearized DNA, and the mRNA was purified using RNeasy spin columns and stored at −80 C.

### Magnetic microspheres for active microrheology

Superparamagnetic Dynabeads M270, 2.8 μm diameter, were used for magnetic microrheology (Invitrogen). The microspheres with streptavidin surface chemistry were passivated with a combination of PEG-biotin (MW 5 K) to prevent non-specific binding and with Atto 550-biotin (Sigma) for visualization in a ratio of 75:25, respectively. Beads were rinsed with PBS and incubated for 30 minutes in excess and then washed several times to remove the reaction mixture. Beads were vortexed each time before use.

### Microinjection and GV dissection

Microinjection and GV dissection were performed according to previously described methods^36^. Briefly, Stage V-VI oocytes were injected with mRNA constructs in the cytoplasm and/or injected with bead solution in the nucleus. After overnight incubation at 18 °C, nuclei were manually dissected in mineral oil under *in vivo* conditions and placed into an appropriate imaging chamber. For active microrheology experiments, oocytes were placed in a PDMS chamber, and for all other experiments, oocytes were placed in between a coverslip and a slide separated by a 1-mm thick silicone well (Grace Bio-labs).

### Centrifugation experiments

Oocytes were placed in OR2 in 1.5 ml Eppendorf tubes and oriented so that the animal pole, containing the nucleus, was on top. Oocytes were spun in a table-top centrifuge (Eppendorf) at 100 g (10, 20, 30, 50 or 100 minutes) or 1,000 g (2, 5, 10, or 20 minutes) or in a centrifuge (ThermoScientific) at 10 g (20 minutes, 3 hours, 8 hours, and 14 hours). For some 1,000 g experiments, the nucleus was pre-dissected and then placed in mineral oil in a 1.5 ml Eppendorf tube and spun as described.

### Electromagnetic tweezers for active microrheology

Electromagnetic tweezers consisted of two opposing pole pieces positioned 5 mm apart. Each cylindrical pole piece was made of annealed MuMetal (The MuShield Company) and was 15 cm in length and 6 mm in diameter. The tip was fabricated to be at a 45° angle and beveled to have a flat edge. The pole pieces were each placed inside a brass-housing unit that was wound with 500 turns of magnet wire (25 AWG, Remington Industries). Electromagnets were connected to a programmable power supply DLM 20–30 (Ametek). The current was directed to each electromagnet via a relay (Seco-Larm). The current and relay were controlled using a custom-built LabVIEW program and a data acquisition device (National Instruments). The analog signal from the power supply was sent to a TTL box, so that the signal was recorded with each image captured during the time-lapse movie using Slidebook Software.

The force as a function of current and the phase shift were determined according to a calibration of the superparamagnetic beads in a glycerol-water solution of measured viscosity using Stokes law. Measured forces produced by the electromagnet could be varied from 0.2 pN to 1.75 pN. Samples were placed in PDMS wells that were bonded to a glass coverslip using plasma treatment or in silicone wells adhering to glass coverslips. During these experiments, the untreated nuclei injected with magnetic microspheres were maintained in a near-physiological condition using mineral oil, as previously described^52^.

### Active microrheology

Using the electromagnetic tweezers, a programmable current was applied to generate a sinusoidal force. A time-lapse movie captured the position of the bead in response to the applied force. The position of the bead as a function of time was fit to the equation 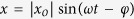, where 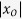 is the amplitude of the bead’s displacement, 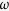 is the angular frequency, 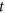 is time, and 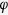 is the phase shift between the bead’s response and the applied force. The storage and loss moduli were determined as a function of frequency from the fit as:


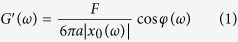



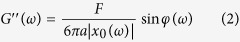


where 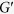 is the storage modulus, 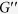 is the loss modulus, 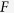 is the amplitude of the force, 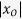 is the amplitude of the bead’s displacement, 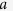
is the bead radius, and 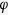 is the phase shift between the bead’s response and the applied force^43^.

In addition to an oscillatory force, a step force was applied to examine the creep response. The time-dependent creep compliance, 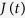, was determined to be^43^:


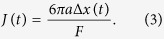


A viscoelastic model that best fit the creep response was a Kelvin-Voigt body in series with a dashpot, which represents long-term viscous flow. The creep compliance for this model is:


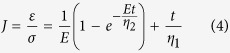


where 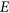 is the elastic modulus of the spring and 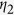 is the viscosity of the dashpot in the Kelvin-Voigt body, while 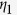 is the viscosity of the dashpot representing long-term flow. The viscoelastic moduli can be obtained by applying a Fourier transformation and were determined for the storage modulus, 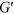, and the loss modulus, 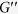, respectively:


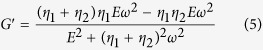



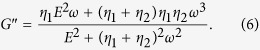


The oscillatory data were fit simultaneously to the above equations for storage modulus, 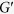, and the loss modulus, 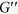, by minimizing the weighted sum of squared residuals.

### Microscopy

Microrheology experiments were performed on an inverted Zeiss spinning-disc confocal microscope as previously described^36^. Briefly, time-lapse experiments were performed using 20X dry and 40X dry objectives at least 20 μm above the coverslip. A Leica SP5 laser scanning confocal microscope was used to obtain high-resolution images of the actin network and nucleoli with a 100X/NA1.46 oil immersion objective. Three-dimensional 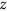-stacks with 2 μm step size were imaged on a custom built two-photon laser scanning system using an upright Olympus BX51W1 microscope with a 40X/NA0.8 water immersion objective lens, a custom-built NA1.4 oil immersion condenser, an objective piezo nano-positioner (Physik Instrumente), photomultiplier tubes (Hamamatsu), and controlled by ScanImage, version 3.8, software.

### Image analysis

Custom-built Matlab software was used for quantitative image analysis. Centroid detection and particle tracking were adapted from Matlab Multiple Particle Tracking Code (see http://physics.georgetown.edu/matlab/index.html)^40^ and used in both active microrheology experiments and centrifugation experiments. A Gaussian fit was applied to the brightest plane of each nucleolus, and nucleolar size was determined to be the half-max full width of the Gaussian fit. The overall shape of the nucleus was determined by applying a threshold filter to each 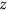-plane to segment the fluorescence of the nucleoplasm from the background. For each 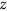-plane, the center and area were determined. Subsequently, the 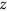-planes were linked together to obtain a 3-D reconstruction of the entire nucleus, which was later fit as an ellipsoid. To regain the sphericity of the nucleus, the nucleus was transformed into a sphere, and this same linear transformation was applied to the coordinates of all the identified nucleoli. The nucleus and nucleoli were rotated by a linear transformation to orient along the apparent gravitational (*z*) axis of the nucleus, which we identified by the vector connecting the centroid of the nucleus with the average position of nucleoli. The distance between the average 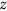-position of nucleoli, 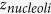, and the centroid of the nucleus, 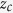, was obtained: 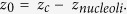 The number density of the nucleoli along the defined z-dimension was determined in bins of twenty microns and a probability distribution was determined for nucleolar size in logarithmically spaced bins.

### Statistical analysis

Error bars were reported as standard error of the mean where number of independent observations, 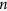, was chosen as the number of nuclei analyzed per experiment. The correlation coefficient for degree of asymmetry (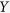) versus nucleolar radius (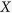) was determined as 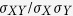. A test statistic was performed at the 95% confidence interval, and the correlations were determined to be statistically significant. Furthermore, 95% confidence intervals for the correlation were determined as 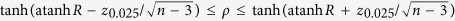, where 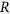 is the sample correlation coefficient^53^.

### Brownian dynamics simulation

We model the gravitational sedimentation and coarsening of nuclear bodies with a simple Brownian dynamics simulation. For simplicity we describe the nucleus by a 1600 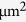 2D square box with reflecting boundary conditions. To mimic the initial density and distribution of nuclear bodies in the nucleus, we initialize the simulation by placing 1,000 particles randomly in this 2D box. The initial volume, 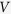, of each particle is randomly chosen from a distribution in which 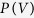 ~ *V*^−1.5^, consistent with our experimental observations (Fig. 4g). The dynamics of the nuclear bodies in the *x-z* plane are simulated using discrete Langevin equations,


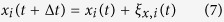



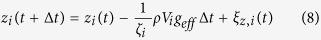


where 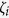 is the effective drag coefficient of particle 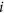 with volume 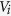 and density 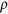, and Δ*t* is the integration time for the numerical Brownian dynamics algorithm. We choose Δ*t* to be small enough such that the typical particle displacement resulting from a single iteration of our algorithm will be smaller than the diameter of the particle. The effective drag coefficient is determined by Stokes law 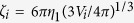, where 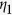 represents the long-time viscosity in the extended Kelvin-Voigt model, which we measured for nuclear actin as described in the main text. The effective gravitational acceleration 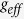 only acts in the *z*-direction. The terms 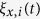 and 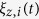 represent the Brownian fluctuations of the particles, which are independently drawn from a Gaussian distribution with zero mean and variance 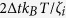 at each time step, where 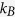 is Boltzmann’s constant and 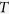 is the temperature. All parameters in our Brownian dynamics model are directly measured by microrheology or quantitative imaging. To model the coarsening dynamics of nuclear bodies, we allow fusion between particles in our simulation. When two nuclear bodies collide with each other, they fuse to become one large body with the combined volume of the initial particles.

## Additional Information

**How to cite this article**: Feric, M. *et al*. Soft viscoelastic properties of nuclear actin age oocytes due to gravitational creep. *Sci. Rep*. **5**, 16607; doi: 10.1038/srep16607 (2015).
